# Progress of non‐motor symptoms in early‐onset Parkinson's disease

**DOI:** 10.1002/ibra.12180

**Published:** 2024-10-09

**Authors:** Fanshi Zhang, Aidi Luo, Shusheng Liao, Mei Liu, Jun Zhang, Zucai Xu

**Affiliations:** ^1^ Department of Neurology Affiliated Hospital of Zunyi Medical University Zunyi China; ^2^ The Collaborative Innovation Center of Tissue Damage Repair and Regeneration Medicine Zunyi Medical University Zunyi China

**Keywords:** autonomic dysfunction, early‐onset Parkinson's disease, late‐onset Parkinson's disease, neuropsychiatric symptoms, non‐motor symptoms

## Abstract

Parkinson's disease (PD) is a common degenerative disease of the central nervous system that is characterized by movement disorders and non‐motor symptoms (NMSs). The associated NMSs primarily include neuropsychiatric symptoms, autonomic dysfunction, sleep‐wake disorders, pain, fatigue, and hyposmia. These NMSs can occur at any stage of PD, especially before the onset of motor symptoms, and may affect a patient's quality of life more than motor symptoms. Although PD is most commonly diagnosed in people over 65 years, some patients exhibit symptom onset before the age of 50, which is clinically known as early‐onset Parkinson's disease (EOPD). The high heterogeneity and incidence of EOPD‐associated NMSs can lead to the misdiagnosis of EOPD as other neurodegenerative diseases. In this review, we discuss the research progress related to NMSs in patients with EOPD, focusing on neuropsychiatric disorders, autonomic dysfunction, sleep disorders, and sensory impairment, and outline the association of NMSs with different genotypic alterations, with the aim of providing assistance in the clinical management of patients.

## Graphical abstract

This study reviews the research progress related to non‐motor symptoms (NMS) in patients with early onset Parkinson's disease (EOPD), including neuropsychiatric symptoms, autonomic dysfunction, sleep disorders, and sensory disorders and also summarizes the characteristics of NMS in the genetic form of Parkinson's disease (PD). Although motor symptoms are the key features of PD, NMS is receiving increasing attention. We believe that our study makes a significant contribution to the literature because this study shows that the NMSs of EOPD and late onset Parkinson's disease are significantly different, mainly in neuropsychiatric symptoms, autonomic function, sleep disturbances, and sensory disturbances. The wide variety and high incidence of NMS in EOPD are often overlooked, and the low rate of recognition and individual differences sometimes make it difficult to distinguish NMS from other neurodegenerative diseases.

Further, we believe that this paper will be of interest to the readership of your journal because it emphasizes the importance of identifying and mastering the characteristics of each NMS in clinical practice for early diagnosis and intervention to improve patients' NMS and quality of life.

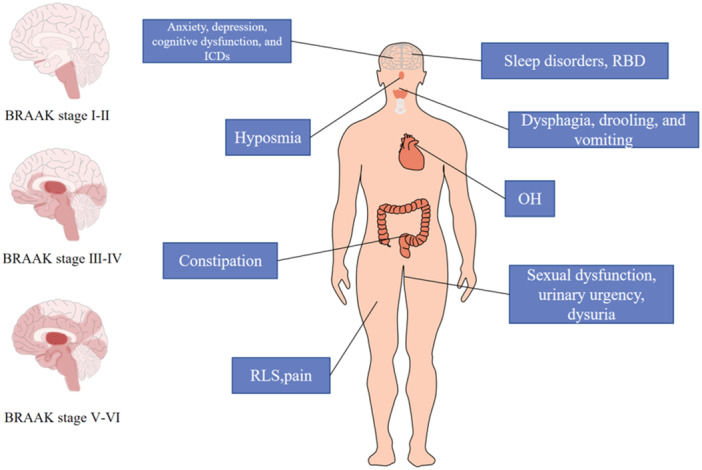

## 1 INTRODUCTION

Parkinson's disease (PD) is a degenerative disorder of the nervous system that develops in middle‐aged and older individuals.^1^ Early‐onset Parkinson's disease (EOPD) generally refers to cases diagnosed at, or before, the age of 50 years, whereas late‐onset Parkinson's disease (LOPD) refers to cases diagnosed after the age of 50 years.^2^ The global incidence of EOPD is 0.81 per 100,000,^3^ with genetic factors contributing significantly to the risk of developing the disease.^4^ Aside from the age difference at diagnosis, EOPD has a slower progression, longer disease duration, higher clinical heterogeneity, and superior response to dopaminergic medications than LOPD. On the other hand, the incidence of levodopa‐induced dyskinesia and symptom fluctuation is higher in patients with EOPD.^5^ Although motor symptoms are key features of PD, research attention is increasingly directed to non‐motor symptoms (NMSs),^6^, ^7^ which primarily include neuropsychiatric symptoms (e.g., depression and anxiety), autonomic dysfunction (e.g., orthostatic hypotension and constipation), sleep disorders (e.g., insomnia and rapid eye movement sleep behavior disorder [RBD]), and sensory disorders (e.g., hyposmia and pain).^8^ Olfactory disturbances, constipation, depression, and RBD may occur in the early stage of PD or before the onset of motor symptoms and may be helpful in diagnosing EOPD.^9^ This review summarizes the research progress on NMSs associated with EOPD to provide a theoretical resource that will aid the clinical diagnosis and management of NMSs in patients with EOPD.

## 2 THE PATHOLOGICAL BASIS OF NMSS IN PATIENTS WITH PD

**Figure 1:**
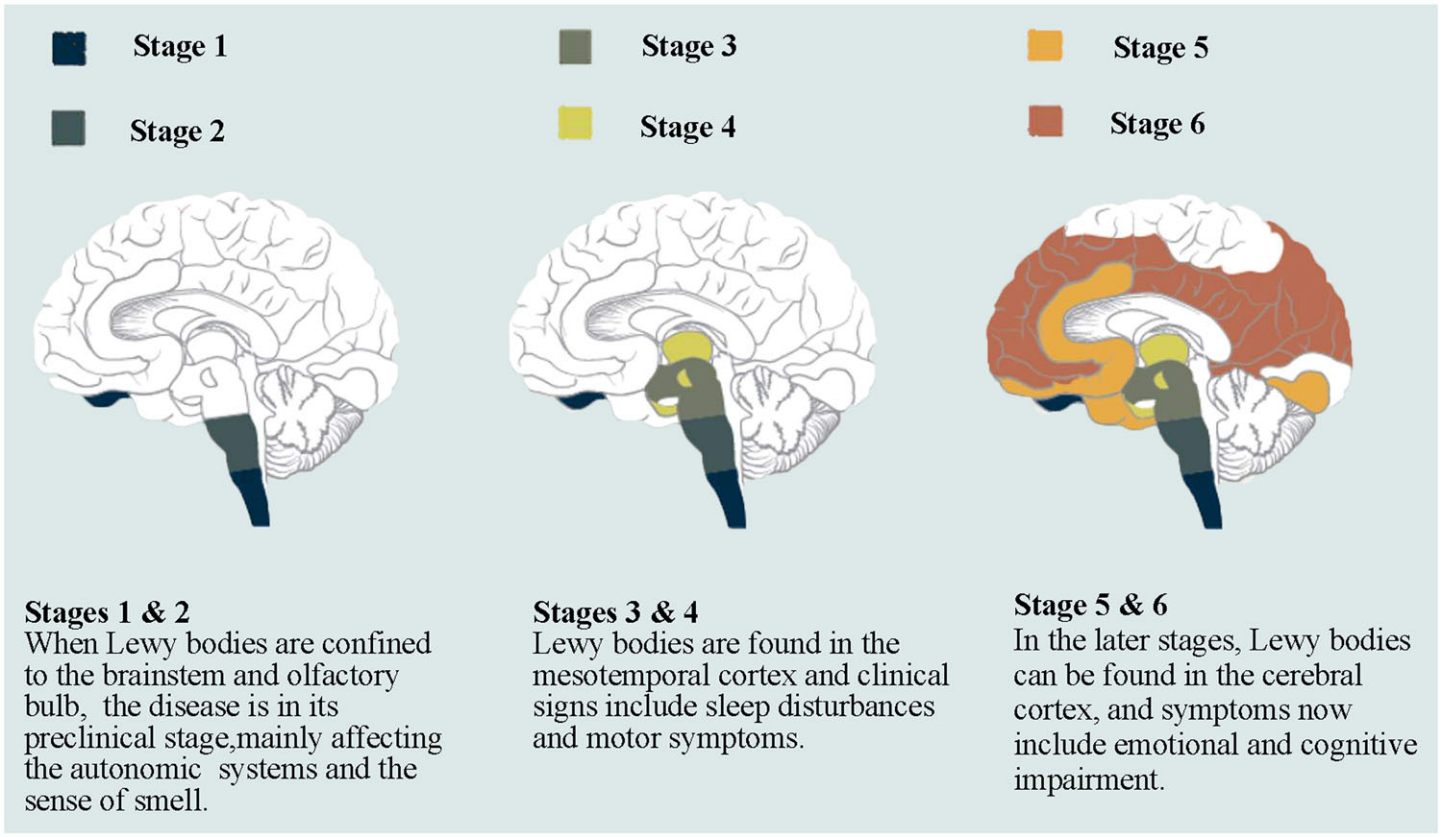
Pathological staging and classification of non‐motor symptoms in patients with early‐onset Parkinson's disease. Braak system is divided into six stages. In Stages I and II, Lewy bodies are confined to the brainstem and olfactory bulb, the disease is in its preclinical stage, mainly affecting the autonomic systems and the sense of smell. In Stages III and IV, Lewy bodies are found in the mesotemporal cortex, and clinical signs include sleep disturbances and motor symptoms. In Stages V and VI, Lewy bodies can be found in the cerebral cortex, and symptoms include emotional and cognitive impairment. [Color figure can be viewed at wileyonlinelibrary.com]

In 2003, Braak et al.^10^ proposed a six‐stage pathological system for the classification of NMS development in PD (Figure 1). Stage I involves pathology in the olfactory bulb, anterior olfactory nucleus, dorsal nucleus of the vagus nerve, and glossopharyngeal nerve,^11^ and the associated deficits include olfactory disturbances and constipation.^12^ Stage II involves pathological findings in the lower brainstem (locus coeruleus, midline raphe nucleus, middle reticular formation, and nucleus accumbens), with symptoms including dysphagia, autonomic dysfunction, and sleep disturbance fatigue.^13^ Stages III and IV are characterized by pathology in the substantia nigra, deep midbrain nuclei, and the telencephalon, and the associated symptoms include resting tremors, bradykinesia, myotonia, and other typical PD motor symptoms.^14^ In stages V and VI, the pathology spreads in the limbic system and neocortex, with manifestation of neuropsychiatric symptoms such as cognitive dysfunction and visual hallucinations.^15^ Hence, NMSs may appear before motor symptoms and gradually worsen as the disease progresses. However, it has also been suggested that the development of NMSs in patients with PD does not correlate with motor symptoms.^16^ Therefore, the Braak staging system requires further confirmation in clinical studies.

## 3 NMSS ASSOCIATED WITH IDIOPATHIC EOPD

### 3.1 Neuropsychiatric symptoms

Neuropsychiatric symptoms, including depression, anxiety, cognitive impairment, impulse control disorders, apathy, and psychotic disorders, are the main causes of reduced quality of life in EOPD.^17^, ^18^

#### 3.1.1 Depression

Depression is the most common neuropsychiatric symptom in patients with EOPD, with an incidence of 30.3%–54.4%, and can precede the onset of motor symptoms. Its pathogenesis is largely mediated by changes in dopaminergic, noradrenergic, and cholinergic neurons.^19^ Brain magnetic resonance imaging (MRI) of patients with PD and depression shows thinning of the cortical gray matter in the limbic system and atrophy in the thalamus and hippocampus, the main site of dopaminergic regulation of mood, motivation, and reward.^20^ Transcranial ultrasound shows hypoechoic, interrupted, or absent midbrain nuclei.^21^ While some studies have suggested that depressive symptoms are not related to age of onset,^22^, ^23^ others have found that patients with EOPD are more likely to experience depressive symptoms.^24^ Compared to LOPD, EOPD is characterized by higher depression scores, more severe depressive symptoms, and higher rates of suicidal ideation.^25^, ^26^ In addition to higher rates of depression, Starkstein et al. found that depression in EOPD is related to cognitive dysfunction and disease course.^24^ In contrast, depression in patients with LOPD is primarily associated with impairment in activities of daily living.^24^

The main anti‐depressant drugs prescribed to patients with PD include selective serotonin reuptake inhibitors, tricyclic antidepressants, and serotonin and norepinephrine reuptake inhibitors.^27^, ^28^ Although tricyclic antidepressants are highly effective in treating depression in these patients, adverse effects, including cardiac arrhythmia, postural hypotension, and urinary retention, are common and must be closely monitored.^27^ Additionally, behavioral cognitive therapy, transcranial magnetic stimulation, and deep brain stimulation have achieved some success in improving depressive symptoms.^29^ In recent years, animal studies have found that autologous transplant therapy alleviates depressive behaviors in parkinsonian monkeys.^30^

#### 3.1.2 Anxiety

Anxiety can occur at any stage in PD and is more common in women and patients with sleep disorders and serious conditions.^31^, ^32^, ^33^ Anxiety can be classified as generalized anxiety disorder, social phobia, nonspecific anxiety disorder, specific phobia, panic disorder, or separation anxiety disorder.^34^ Patients with anxiety symptoms are more likely to present with typical motor symptoms, fluctuations in motor symptoms, and autonomic dysfunction.^35^ Animal models of PD have shown that bilateral nigrostriatal damage, defects in the vesicular monoamine transporter protein 2, and reductions in the levels of dopaminergic, noradrenergic, and 5‐hydroxytryptamine (5‐HT) activity contribute to the development of anxiety.^36^ Despite these observations, data that directly demonstrate a pathological or biochemical basis for anxiety in patients with PD are scarce.^19^

Anxiety is more common in patients with LOPD than EOPD and may affect cognitive dysfunction.^33^ Although anxiety scores negatively correlate with age at PD onset, the difference in the rates of anxiety between patients with EOPD and LOPD is not significant.^25^ Of note, anxiety is consistently accompanied by depression, which is the factor that most significantly affects quality of life in patients with EOPD.^37^

Anxiety symptoms in PD can be improved with anti‐depressant treatment^25^ or by adjusting the dose of anti‐Parkinsonian drugs and administering 5‐HT reuptake inhibitors. However, these strategies can lead to excessive daytime sleepiness, cognitive dysfunction, and gait disturbance.^38^ As an alternative, benzodiazepines may provide short‐term relief of anxiety symptoms, but their use can increase the risk of falls and fractures.^39^

#### 3.1.3 Cognitive dysfunction

Most people with PD have varying degrees of cognitive dysfunction, ranging from mild cognitive impairment (PD‐MCI) to dementia (PD‐D).^9^, ^40^ PD‐MCI progresses to PD‐D in 6%–15% of patients.^9^, ^40^ Advanced age is a risk factor for cognitive dysfunction, with a significantly higher incidence of PD‐MCI and PD‐D in LOPD than in EOPD.^41^, ^42^, ^43^ Compared to Alzheimer's disease, which is characterized by memory impairment, cognitive dysfunction in PD‐D is dominated by visuospatial and executive deficits.^44^, ^45^ More specifically, LOPD is associated with more severe cognitive impairment than EOPD, with significantly lower scores in the mini‐mental state examination and Montreal Cognitive Assessment.^41^, ^46^ In LOPD, executive function can become impaired even at early stages of the disease, while cognitive dysfunction progresses slower in EOPD.^41^

The progression of cognitive impairment in patients with PD is markedly affected by Lewy body pathology and also influenced by dysfunction in the dopamine system.^47^ In animal studies, researchers have found that chronic α‐synuclein (SNCA) accumulation in the rat hippocampus induces Lewy body formation and specific cognitive deficits.^48^ Impaired dopaminergic function in midline cortical pathways is primarily associated with executive dysfunction, whereas impaired function in the noradrenergic locus coeruleus may lead to decreased alertness and cognitive flexibility.^49^, ^50^ Imaging studies have shown that hippocampal atrophy, dopamine function in the caudate nucleus, and cholinergic dysfunction are associated with cognition impairment in PD.^51^ Interestingly, in patients with EOPD, apoptosis of dopaminergic neurons occurs primarily in the nucleus accumbens, whereas, in LOPD, apoptosis is also noted in the caudate nucleus.^52^

Considering that the use of anticholinergic drugs should be minimized, nonpharmacological treatment of cognitive dysfunction primarily includes cognitive training, aerobic exercise, and repetitive transcranial magnetic stimulation.^53^, ^54^ The cholinesterase inhibitors rivastigmine and donepezil have proven effective for the treatment of cognitive impairment, but data regarding their efficacy in PD are lacking.^55^, ^56^

#### 3.1.4 Impulsive–compulsive behavior

The principal manifestations of impulsive–compulsive behaviors in patients with PD include a range of impulse control disorders, such as pathological gambling, internet addiction, hypersexuality, overeating, and compulsive shopping.^57^ Certain confounding factors, including early disease onset, high levodopa dose, dopamine agonist history, alcoholism, young age, and male sex, are strongly associated with the development of impulse control disorders.^58^, ^59^ A cross‐sectional, multi‐center study found that 58.3% of patients with impulse control disorders in EOPD had higher depression scores and poorer quality of life than healthy young people.^60^ Moreover, patients with EOPD who had been administered dopamine agonists had a seven‐fold greater risk of developing impulse control disorders.^60^ Hence, the dose of the dopamine agonist should be reduced or discontinued in these patients while monoamine oxidase‐B (MAO‐B) inhibitors can be added.^61^ In summary, simply lowering the dose of dopaminergic medications is usually helpful in controlling impulse control disorders. Administration of nonpharmacologic treatments such as deep brain stimulation is also based on this principle.^57^, ^62^, ^63^

### 3.2 Autonomic dysfunction

Autonomic dysfunction occurs in up to 50% of PD cases^64^ and involves the cardiovascular, gastrointestinal, urinary, and thermoregulation systems.^65^ It is generally accepted that the degree of autonomic dysfunction is milder in patients with EOPD than in those with LOPD.^1^, ^64^

#### 3.2.1 Cardiovascular regulation disorders

Orthostatic hypotension is the most prominent autonomic dysfunction of the cardiovascular system in patients with PD, with a prevalence of 9.6%–64.9%,^66^ and is mainly associated with degeneration in the cardiac sympathetic system following a disordered pressure reflex, reduced activation of noradrenergic pathways, or vagal dysfunction.^67^, ^68^ Approximately 36% of patients with EOPD are diagnosed with orthostatic hypotension with supine hypertension and those with supine hypertension are at a greater risk of developing orthostatic hypotension.^69^ Accordingly, Espay et al. suggested that patients with PD who develop supine hypertension with orthostatic hypotension should be prioritized for management of the latter due to its higher associated risk.^70^ Treatment of orthostatic hypotension should begin with clinical education, including slow movements on rising, increased fluid intake, avoidance of long‐acting antihypertensive drugs, and elevation of blood pressure in the upright position with midodrine if symptoms are not well controlled.^71^, ^72^

#### 3.2.2 Gastrointestinal dysfunction

Gastrointestinal dysfunctions associated with PD include constipation, dysphagia, salivation, loss of taste, nausea, and vomiting.^73^ In recent years, researchers have increasingly suggested that PD pathology may arise in the gastrointestinal tract and spread through the sympathetic and parasympathetic nervous systems to the substantia nigra and central nervous system (CNS).^74^ For example, in experiments with mice, intragastric administration of the pesticide rotenone almost exactly reproduces the typical pathological and clinical features of PD.^74^ The etiology of gastrointestinal dysfunction in patients with PD is multifactorial and may include deposition of SNCA throughout the enteric nervous system.^75^ Constipation, as one of the most common NMSs appearing before the diagnosis of PD, is often overlooked.^76^ Peripheral nervous system and CNS disorders are associated with decreased peristaltic movements in the colon, muscle tension caused by impaired relaxation of the anal sphincter, and dysfunction of the rectal muscle, all of which lead to a reduced number of bowel movements.^77^ Constipation is less common in patients with EOPD than in those with LOPD since the incidence of constipation in mid‐ and late‐stage PD is higher,^23^ which may be related to older age, longer disease duration, more severe motor symptoms, and the administration of dopamine replacement drugs.^78^ Dopamine replacement drugs such as levodopa and benserazide hydrochloride tablets, carbidopa and levodopa controlled‐release tablets, and anticholinergic drugs can have adverse effects on constipation.^78^ Nonpharmacological treatment modalities include increased intake of water and dietary fiber and exercise. The most common pharmacological treatment options include laxative drugs, such as polyethylene glycol and lactulose, as well as gastrointestinal stimulants, such as domperidone and mosapride.^79^ Additionally, fecal transplantation has proven effective in treating constipation in patients with PD.^80^, ^81^

#### 3.2.3 Urinary and reproductive system dysfunction

The prevalence of urinary and reproductive system dysfunctionin patients with PD is 57%–83%, and may be positively correlated with age and disease duration. Common urinary and reproductive symptoms include sexual dysfunction, urinary frequency and urgency, nocturia, and difficulty urinating.^82^ Sexual dysfunction affects approximately 80% of men and 40% of women with PD.^83^ Guo et al. found that sexual dysfunction is more common in patients with EOPD than in those with LOPD,^84^ but Spica et al. found a significantly higher frequency of altered sexual desire and sexual behavior disorder in LOPD.^33^ Generally, older patients and those with later disease onset are more likely to experience sexual dysfunction. Compared with EOPD, LOPD is associated with a higher rate of sexual function disorders, including reduced libido, erectile dysfunction, and difficulty in achieving orgasm.^85^ In addition, recurrent urinary tract infections were found to lead to progressive development of PD in experiments in mice.^86^

Urinary tract symptoms can occur in the early stages of PD and become more frequent as the disease progresses.^83^ Therefore, compared with patients with LOPD, those with EOPD are less likely to experience frequent urination and nocturia.^85^ The primary treatment for urinary symptoms is behavioral therapy^87^; while anticholinergic drugs can be used to treat overactive bladder dysfunction and sildenafil and dopamine agonists are effective for the treatment of sexual dysfunction.^87^

## 4 SLEEP DISORDERS

Sleep disorders are common in patients with EOPD and can occur at all stages of the disease course, even before the appearance of motor symptoms.^88^ Dopamine dysfunction and sleep/wake alterations have been found to be closely related in mouse models of the disease.^89^ The most common sleep disorders associated with EOPD include insomnia, excessive daytime sleepiness, restless leg syndrome, RBD, obstructive sleep apnea, and circadian rhythm disturbances.^90^ The origins of sleep disorders are multifactorial and related to age, severity of illness, substance use, excessive nocturia, and depression, and the primary causes include degenerative lesions in the brainstem sleep regulation centers and disruptions in thalamocortical pathways.^91^

Insomnia and excessive daytime sleepiness are reportedly the main manifestations of sleep disorders in EOPD and LOPD. Patients with EOPD report better sleep quality, less difficulty in maintaining sleep, and less frequent daytime sleepiness than those with LOPD.^92^, ^93^ Sleep disturbances can be affected by cognitive function in patients with EOPD and LOPD, whereas sleep difficulty is affected by the total daily levodopa dose in patients with EOPD, and by depression and anxiety status in those with LOPD.^94^

Behavioral cognitive therapy, chronotherapy, relaxation therapy, and phototherapy may improve insomnia symptoms.^95^ Administration of dopamine agonists (e.g., ropinirole and pramipexole extended‐release tablets) may improve sleep quality by maintaining dopaminergic blood levels. Additionally, the MAO‐B inhibitor rasagiline may improve nocturnal tremor symptoms, thereby improving sleep onset.^96^, ^97^ In addition, dexzopiclone and melatonin may be helpful in improving sleep quality.^96^

### 4.1 RBD

The incidence of RBD in patients with PD is 46%–58% and is characterized by skeletal muscle dystonia and dream enactment behaviors during rapid eye movement sleep, including yelling, body movements, sleep talking, and aggressive behavior.^98^ The mechanism of loss of muscle atonia may involve the deposition of SNCA in a pontine nucleus and is associated with lesions in the sublaterodorsal nucleus, lateral portion of the pontine tegmentum, ventrolateral periaqueductal gray, and the locus coeruleus, as well as other sites between brainstem nuclei and the nucleus basalis.^99^ In patients with suspected RBD, the RBD screening questionnaire and RBD questionnaire in Hong Kong are commonly used for initial clinical screening, while polysomnography is applied to verify the diagnosis.^100^ Although the symptoms of RBD generally appear 10 years before the onset of PD motor symptoms, the incidence of RBD is lower in patients with EOPD than in those with LOPD.^101^, ^102^ Moreover, patients with RBD progress more rapidly and have a higher risk of dementia compared to patients without RBD.^98^, ^101^, ^102^ Low doses of clonazepam and melatonin at bedtime can be effective in treating RBD, although clonazepam can increase the risk of falls. ^103^, ^104^, ^105^ In recent years, PD mouse models with RBD‐related behaviors have been established, and more treatment options are worth exploring in the future.^106^

### 4.2 Restless leg syndrome

The prevalence of restless leg syndrome in patients with treated PD is 15% and 11% in patients with untreated PD.^107^ The primary manifestation of the syndrome is unbearable discomfort in the limbs of patients at rest or during nighttime sleep, which must be relieved by moving the limbs.^108^ The restless leg syndrome is primarily caused by impaired cerebral iron metabolism and degeneration of dopaminergic neurons.^109^ Although several studies have found that it occurs more commonly in EOPD, the Chinese Parkinson's Disease Registry study reported that the incidence of restless leg syndrome in EOPD is limited compared to that in LOPD^2^, ^101^, ^110^

Alcohol or caffeine intake, stress, and administration of antidepressants, dopamine antagonists, or antihistamines may increase the risk of developing restless leg syndrome and should be minimized or discontinued.^111^ Iron supplementation is recommended for patients with serum ferritin levels <50–75 µg/L,^111^ and administration of a long‐acting dopamine agonist and calcium channel alpha2delta ligand analog at bedtime may improve symptoms.^98^ In recent years, researchers have increasingly focused on the emergence of restless leg syndrome in primate models of hyperactivity disorder and in nonprimate animal models in an attempt to open up new therapeutic directions.^112^

## 5 SENSORY IMPAIRMENT

Patients with EOPD are prone to sensory symptoms, including olfactory and visual impairment and pain, with the latter being more prevalent in patients with EOPD than in those with LOPD.^113^ As a prodromal symptom of PD, the incidence and severity of sensory disorders can progress with the disease course.^114^

### 5.1 Olfactory disorders

Olfactory disturbances can precede motor symptoms in PD and are categorized as a potential “warning sign” of PD, according to the 2013 PD diagnostic guidelines of the European Federation of Neurological Societies.^115^ Clinical assessment strategies commonly include subjective examination methods that directly assess olfactory function (olfactory discrimination, threshold, and memory) as well as olfactory electrophysiological testing and neurofunctional imaging.^116^ In particular, identification of coffee, mint, and aniseed odors is consistent with the subjective olfactory function testing results and is beneficial to olfactory testing for clinical screening in EOPD.^117^

The pathogenesis of olfactory disorders involves deposition of Lewy bodies in the olfactory bulb, anterior olfactory nucleus, olfactory tract, and the piriform cortex. Hyposmia or loss of smell occurs in >90% of patients with PD, but these signs are often overlooked.^118^ Other common olfactory impairment manifestations include decreased odor discrimination, odor memory, and perceptual threshold, which are more severe in men.^119^ Although the incidence of olfactory impairment is high in PD, the olfactory function is not related to disease duration, score in the unified Parkinson's disease rating scale III, or levodopa dosage.^120^ Moreover, patients with EOPD who develop the disease before the age of 45 have better retention of smell, while those with LOPD are more likely to develop olfactory disturbances.^22^, ^121^ In a mouse model, olfactory impairment also emerged as an important precursor for detecting the onset of PD.^122^

### 5.2 Pain

Approximately 76% of patients with PD experience pain; in fact, severe pain symptoms can mask motor symptoms.^123^ Skeletal muscle pain is the most universal type of pain in patients with PD and fluctuates with patient's motor status, typically worsening in the “off” state.^124^ The pathogenesis of pain may be related to the enhanced processing of pain signals by the CNS, lower pain threshold, altered pain‐evoked potentials, and pain‐evoked increased activation in cortical networks.^125^, ^126^ In EOPD, pain is more severe, with a high incidence,^127^ and is commonly experienced in the lower back and lower limbs, whereas patients with LOPD tend to report pain in the lower limbs and joints.^78^ Although pharmaceutical interventions are not required for mild symptoms, optimized treatment strategies, including dopaminergic therapy, physiotherapy, opioids, and deep brain stimulation can be beneficial in relieving pain.^128^, ^129^ Research in a rat model of PD revealed that nondopaminergic circuits are critical for the development of NMSs, especially for pain, and may be a new direction for the future treatment of NMSs in PD.^130^

### 5.3 Visual disorders

Visual disorders in patients with PD can occur at different locations along the pathway from the retina to the visual cortex and can involve the oculomotor system. These disorders primarily include light adaptation adjustment disorder, blurred vision, and impairment in color discrimination and motion perception.^131^, ^132^ Similar effects have been validated in primate models.^133^ Patients with EOPD often present with various ocular and visual dysfunctions, including abnormalities in smooth pursuit and visual sensitivity, dry eye, and visual hallucinations.^132^, ^134^ These symptoms are often overlooked or ignored during early stages of disease, resulting in delayed diagnosis or progressive PD and increased disability.^134^ A comparison in the sensitivity and color vision ability between patients with EOPD or LOPD and age‐matched controls revealed that visual loss occurred in both patient groups but was more pronounced in EOPD, consistent with the idea that NMSs are influenced by age at symptom onset.^135^ Nevertheless, additional studies on the visual function in patients with EOPD and LOPD are needed to understand the relationship between aging and the pathophysiology of non‐motor PD symptoms to facilitate early diagnosis and, potentially, better management of the disease.^135^ Indeed, clinical attention should be targeted to ocular and visual symptoms in patients with PD to ensure a rational treatment plan is developed.^135^

**Figure 2:**
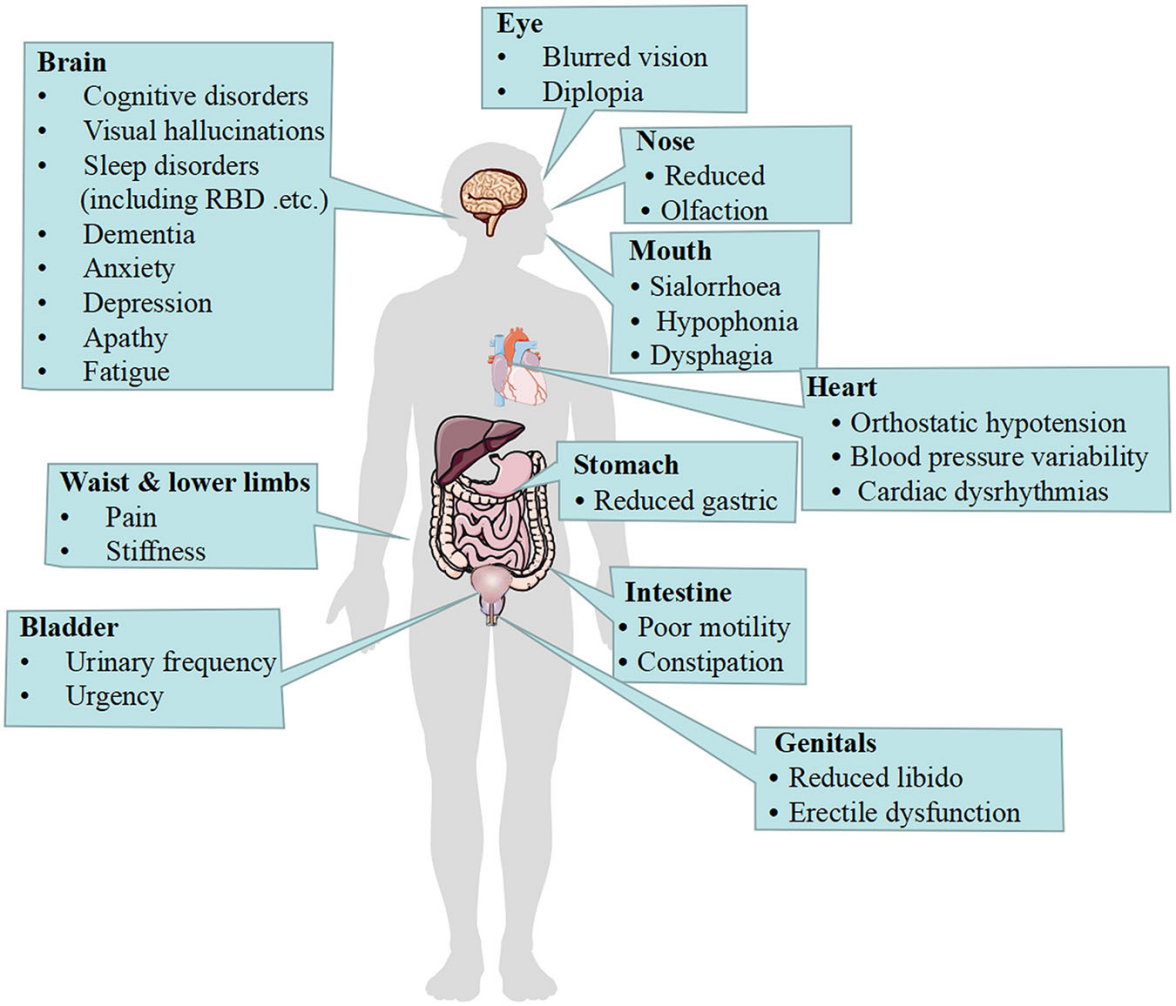
Non‐motor symptoms in patients with early‐onset Parkinson's disease. As the disease progresses, multi‐system involvement often occurs, manifesting as neuropsychiatric symptoms, autonomic disorders, sleep disorders, and sensory disturbances, etc. [Color figure can be viewed at wileyonlinelibrary.com]

**Figure 3:**
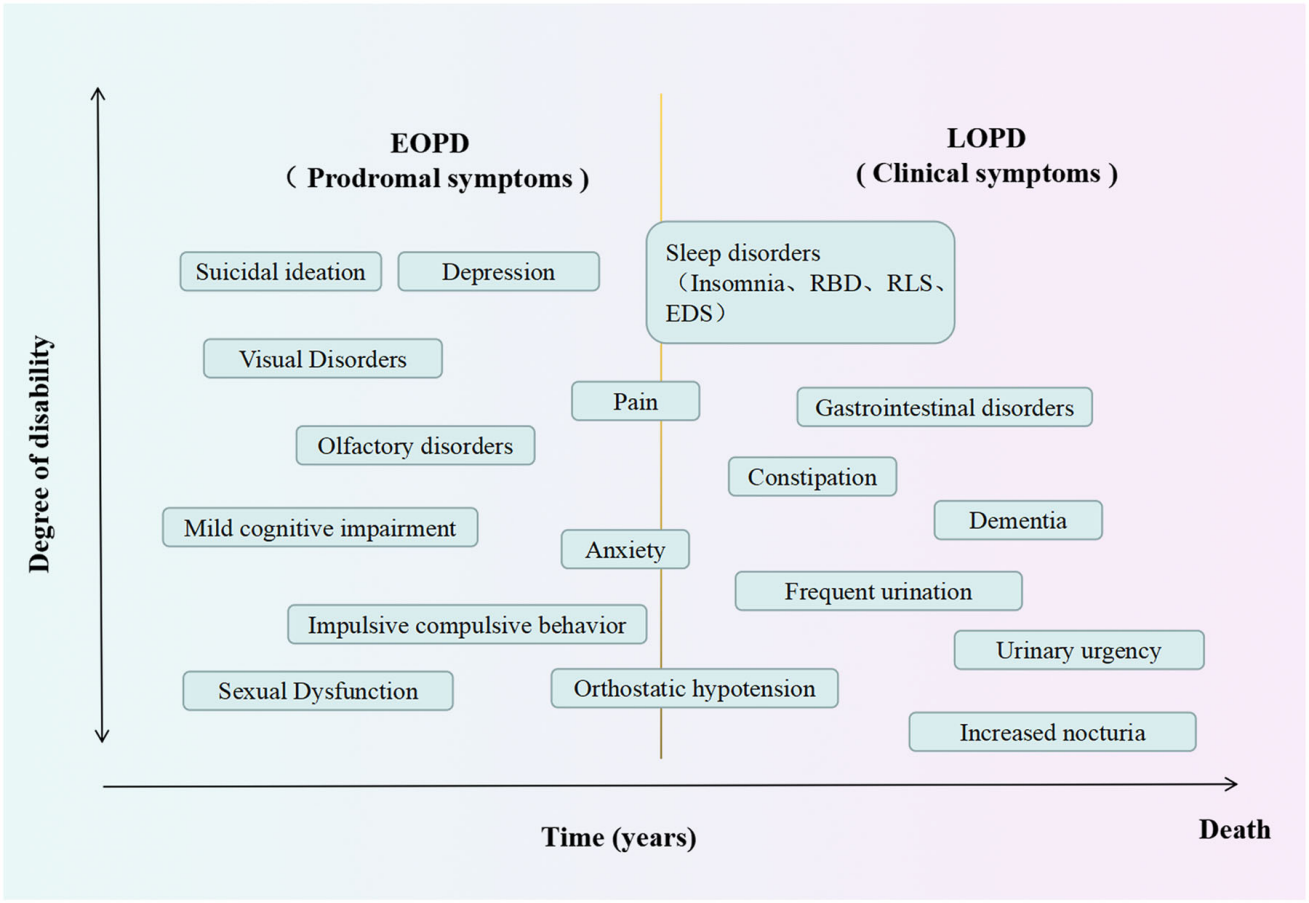
Differences in non‐motor symptoms between early‐onset Parkinson's disease (EOPD) and late‐onset Parkinson's disease (LOPD). Patients with EOPD may be more likely to first experience suicidal ideation, depression, olfactory and visual disturbances, mild cognitive impairment, impulsive compulsive behavior, and sexual dysfunction. Anxiety and pain are likely to occur among both EOPD and LOPD patients. Patients with LOPD may present more often with sleep disturbances, gastrointestinal disorders, constipation, dementia, frequent urination, urinary urgency, and increased levels of nocturia. [Color figure can be viewed at wileyonlinelibrary.com]

To summarize, Figure 2 outlines the NMSs that may occur in patients with EOPD. Figure 3 illustrates the changes in NMS manifestation over time during PD progression.

## 6 NMSS IN DIFFERENT EOPD GENOTYPES

Approximately 50% of familial and 15% of sporadic EOPD cases are associated with Parkin mutations.^136^ The EOPD phenotype with Parkin mutations is characterized by slower progression of cognitive dysfunction, better sleep quality, and relative olfactory retention; however, depression is more common.^137^, ^138^ Patients with mutations in PTEN‐induced putative kinase 1 (PINK1) and Parkinsonism‐associated deglycase (DJ‐1) exhibit early‐age onset disease and relatively slow progression of motor symptoms, while cognitive dysfunction is rarely observed.^139^ On the other hand, glucosylceramidase beta (GBA) mutations are associated with more severe motor decline and myotonia and the patients are more likely to experience NMSs, such as cognitive dysfunction, depression, olfactory disturbances, and RBD.^140^ Most patients with SNCA gene mutations present with rapidly progressive cognitive dysfunction, while a minority have neuropsychiatric symptoms.^141^ Patients with phospholipase A2 group VI (PLA2G6) mutations consistently exhibit dystonia–parkinsonian syndrome at early‐age onset and comorbidities with neuropsychiatric symptoms.^142^ ATPase cation transporting 13A2 (ATP13A2) mutations are associated with early‐age onset, neuropsychiatric symptoms, and cognitive dysfunction, among other symptoms.^143^ However, NMSs, such as hyposmia, are relatively uncommon in patients with leucine‐rich repeat kinase 2 (LRRK2) mutations.^144^

The development of PD is associated with the formation of Lewy bodies.^145^ SNCA exists in a monomeric state in unaffected individuals, while, in patients with PD, these monomers aggregate into synuclein complexes, which, together with other proteins, form Lewy bodies that precipitate in the brain. In this process, microglia were activated, with subsequent release of inflammatory mediators and exacerbation of neuronal damage.^146^ In rare cases, PD may not produce Lewy bodies,^147^, ^148^ for example, due to Parkin mutations.^148^, ^149^, ^150^ Recessive forms of PD are not associated with Lewy body disease and, consequently, NMSs, which feature in dominant forms or sporadic PD cases, are absent.^149^, ^150^ In most cases of PD, for example, neuropathologic evaluation of patients with Parkin mutations do not show Lewy body pathology, although exceptions have been observed.^148^, ^149^, ^151^ In addition, the pathology in a small number of patients with PD with PINK1 mutation lacked Lewy bodies.^152^

## 7 DISCUSSION

PD is characterized by clinically heterogeneous symptoms across several aspects, such as motor symptoms and NMSs, disease progression, and responsiveness to dopamine treatment, among patients with different ages of onset.^153^ LOPD tends to initially present with a combination of clinical signs such as tremors, increased muscle tone, and slow movements.^154^, ^155^, ^156^ In contrast, EOPD commonly presents with atypical symptoms, including foot dystonia, longer disease course, slower progression, and more effective response to levodopa.^154^, ^155^, ^156^ The occurrence of NMSs correlates with various factors, including patient age, disease duration and severity, medication, and genetics.^16^ with the latter exerting a greater influence on the occurrence of EOPD than LOPD.^157^ In fact, first‐degree relatives of patients with EOPD exhibit NMSs more frequently, and their risk of developing PD is higher than that for relatives of patients with LOPD.^158^ Table 1 showcases the main references and findings that demonstrate the differences between EOPD and LOPD.

**Table 1:** Study characteristics. It showcases the main references and findings that demonstrate the differences between EOPD and LOPD.

Author	Year	Country	Type of Study	Design of Study	Results
Starkstein et al.^24^	1989	USA	Human	RCT	Depression is the most common neuropsychiatric symptom in patients with EOPD, with an incidence of 30.3%–54.4%, and can precede the onset of motor symptoms.
Ou et al.^26^	2021	China	Human	RCT	Compared to LOPD, EOPD has higher depression scores, more severe depressive symptoms, and higher rates of suicidal ideation.
Spica et al.^33^	2013	Serbia	Human	RCT	Anxiety is more common in patients with LOPD than EOPD and may affect cognitive dysfunction. Otherwise, the study also found a significantly higher frequency of altered sexual desire and sexual behavior disorder in LOPD.
Fereshtehnejad et al.^25^	2014	Iran	Human	RCT	Although anxiety scores are negatively correlated with age at PD onset, the difference in rates of anxiety between EOPD and LOPD patients is not significant.
Walter et al.^37^	2007	Germany	Human	RCT	Anxiety is consistently accompanied by depression, which is the factor that most significantly impacts EOPD quality of life
Tang et al.^41^	2016	China	Human	RCT	Advanced age is a risk factor for cognitive dysfunction, with the incidences of PD‐MCI and PD‐D significantly higher in LOPD than in EOPD.
Wickremaratch et al.^46^	2009	UK	Human	Systematic review	LOPD is associated with more severe cognitive impairment with significantly lower MMSE and MoCA scores than EOPD.
Vela et al.^60^	2016	Spain	Human	RCT	A cross‐sectional, multi‐center study found that 58.3% of patients with ICDs in EOPD had higher depression scores and poorer quality of life, which was significantly higher than that in healthy young people.
Merola et al.^64^	2017	USA	Human	RCT	It is generally accepted that the degree of autonomic dysfunction is milder in patients with EOPD compared with LOPD.
Yoritaka et al.^69^	2020	Japan	Human	RCT	Approximately 35.88% of patients with EOPD are diagnosed with OH and supine hypertension; also, those with supine hypertensionare at a greater risk of developing OH.
Fasano et al.^77^	2015	Canada	—	Review	Peripheral and CNS disorders cause reduced peristaltic movement of the colon, muscle tension caused by impaired relaxation of the anal sphincter, and dysfunction of the rectal muscle, all of which lead to a reduced number of bowel movements.
Zhou et al.^23^	2013	China	Human	RCT	The incidence of constipation in mid‐ and late‐stage PD is higher, which may be related to older age, longer disease duration, more severe motor symptoms, and administration of dopamine replacement drugs.
Guo et al.^84^	2013	China	Human	RCT	The study found that sexual dysfunction is more common in patients with EOPD than LOPD.
Özcan et al.^85^	2016	Turkey	Human	RCT	Compared with EOPD, LOPD is associated with a higher rate of sexual function disorders, including reduced libido, erectile dysfunction, and difficulty achieving orgasm.
Mahale et al.^93^	2015	India	Human	RCT	Insomnia and EDS are reportedly the main manifestations of sleep disorders in EOPD and LOPD. However, patients with EOPD have better sleep quality, less difficulty in maintaining sleep, and less daytime sleepiness than those with LOPD.
Jurcau and Vharoon.^94^	2021	Romania	Human	RCT	Sleep disturbances can be affected by cognitive function in patients with EOPD and LOPD, whereas sleep difficulty is affected by the total daily levodopa dose in patients with EOPD, and by depression and anxiety in those with LOPD.
Zhou et al.^101^	2022	China	Human	RCT	Although the symptoms of RBD generally appear 10 years before the onset of PD motor symptoms, incidence of RBD is lower in patients with EOPD than LOPD.
Zhou et al.^102^	2022	China	Human	RCT	Patients with RBD progress more rapidly and have a higher risk of dementia compared to patients with PD without RBD.
Calne et al.^110^	2008	Canada	—	Review	Although several studies have found that RLS occurs more commonly in EOPD, the Chinese Parkinson's Disease Registry study reported that the incidence of RLS in EOPD is limited compared to that in LOPD.
Santos et al.^113^	2010	España	—	Review	Patients with EOPD are prone to sensory impairment symptoms, including olfactory impairment, pain, and visual impairment. Of which, pain is more prevalent in patients with EOPD than LOPD.
Santin et al.^121^	2010	Brazil	Human	RCT	Moreover, patients with EOPD who develop the disease before the age of 45 years have better retention of smell, while those with LOPD are more likely to develop olfactory disturbances.
Rodríguez‐Violante et al.^78^	2016	Mexico	Human	RCT	In EOPD, pain is more severe, its incidence is higher, and is commonly experienced in the waist and lower limbs, whereas patients with LOPD tend to report pain in the lower limbs and joints.
Zarkali et al.^132^	2022	UK	Human	RCT	Patients with EOPD often present with various ocular and visual dysfunctions, including sweeping dysfunction, visual sensitivity, dry eye, and visual hallucinations.
Feitosa‐Santana et al.^135^	2020	Brazil	Human	RCT	A comparison of the sensitivity and color vision in EOPD and LOPD patients with age‐matched controls revealed that visual loss occurred in both patient groups, however, was more pronounced in EOPD, consistent with the idea that NMSs are influenced by age at symptom onset.

Abbreviations: CNS, central nervous system; EDS, excessive daytime sleepiness; EOPD, early‐onset Parkinson's disease; ICDs, impulse control disorders; LOPD, late‐onset Parkinson's disease; MMSE, mini‐mental state examination; MoCA, Montreal Cognitive Assessment; NMSs, non‐motor symptoms; OH, orthostatic hypotension; PD, Parkinson's disease; PD‐D, dementia; PD‐MCI, mild cognitive impairment; RBD, rapid eye movement sleep behavior disorder; RCT, Randomized Controlled Trial; RLS, restless legs syndrome; UK, The United Kingdom; USA, The United States of America.

NMSs are prevalent at all stages of PD; the longer and more severe the disease, the greater the number of NMSs. NMSs in the prodromal phase of PD primarily include olfactory disturbances, constipation, depression, and RBD, which may prove useful in the early diagnosis of PD.^19^ Overall, patients with EOPD have a high risk of developing NMSs; however, the fact that most patients with EOPD are often responsible for supporting their parents and children while facing professional and life challenges as well as the financial burden of long‐term medication,^26^ might account for the higher rates of depression, more serious depression, and significantly higher levels of suicidal ideation compared to LOPD.^26^ Some studies have found that patients with LOPD are more likely to experience anxiety and depression, which may be related to disease severity.^33^ Patients with EOPD often retain a relatively good sense of smell compared to patients with LOPD. Considering that olfactory impairment correlates with cognitive function, olfactory impairment may predict an increased risk of cognitive decline.^159^ Regarding sexual dysfunction in EOPD, although the findings are inconsistent, in general, impaired sexual function is more common in patients with LOPD, who also experience more frequent urinary symptoms, including urinary frequency, urgency, and difficulty in urinating.^84^, ^85^ The main manifestations of sleep disorders in EOPD and LOPD are insomnia and excessive daytime sleepiness, while patients with EOPD are more likely to present with restless leg syndrome but better overall sleep quality.^160^ Overall, the most universal NMSs in EOPD are depression, restless leg syndrome, pain, and impulse control disorders, while cognitive and urinary dysfunction and hallucinations are relatively rare and olfactory function is relatively well preserved.^23^ LOPD often presents with more severe and a greater range of NMSs, including sleep disturbances, cognitive dysfunction, gastrointestinal and urinary symptoms, and a poorer quality of life than EOPD.^32^ Thus, NMSs in EOPD and LOPD differ significantly, primarily with regards neuropsychiatric symptoms, autonomic function, sleep disturbances, and sensory dysfunction.^161^ However, these differences have proven somewhat controversial in different studies, suggesting that a certain level of clinical heterogeneity exists in the presence of NMSs between PD subtypes with different age at onset.^162^

The wide variety and high incidence of NMSs in EOPD are often overlooked, while the low rate of recognition and individual differences can make it difficult to distinguish PD from other neurodegenerative diseases.^27^ Hence, it is important to comprehensively characterize the main clinical characteristics of each NMS to achieve early diagnosis and intervention, thereby improving patients' NMSs and quality of life.^163^ Many hypotheses have been proposed to describe the pathogenesis of PD,^164^ but with a highly complex and not fully understood etiology, the clinical eradication of this disease remains elusive.^165^, ^166^ Accordingly, there is no effective cure available although several treatment options are regularly implemented to manage NMSs in PD. Despite the availability of various pharmaceuticals and rehabilitation modalities, no effective outcomes have been achieved.^167^, ^168^ Current treatments for NMSs include medications, non‐pharmacologic treatments, deep brain stimulation surgery, and functional exercises.^169^, ^170^, ^171^ Nonpharmacological treatments include psychotherapy, electrical nerve stimulation, and hydrotherapy.^171^ In recent years, multidisciplinary interventions have been recommended for the early management of PD‐associated NMSs, such as a multidisciplinary health education program in Spain that involves clinicians, counselors, and nurses. The program has improved the quality of life and psychological adjustment of patients with PD and family caregivers in the community through psychoeducation and health promotion.^172^ Meira et al. used hydrotherapy to intervene in patients with early‐stage PD and showed that, compared to land‐based training, the treatment improved pain symptoms, depression, and quality of life in patients with mild‐to‐moderate PD.^173^ In the future, with the increasing attention to NMSs, a growing number of new methods and comprehensive and intensive therapeutic measures are expected to be explored and applied. Of note, while research in the field of NMSs associated with PD has lagged in China, recent studies by local researchers are expected to lead to improved early detection, diagnosis, and treatment strategies to prolong the course of PD and improve quality of life.

## AUTHOR CONTRIBUTIONS

Fanshi Zhang and Aidi Luo contributed to the conception and design of this study, collected and reviewed the relevant literature. Mei Liu and Shusheng Liao designed the article structure. Fanshi Zhang, Aidi Luo, Jun Zhang, and Zucai Xu wrote the first draft of this manuscript. All authors contributed to manuscript revision and read and approved the submitted version.

## CONFLICT OF INTEREST STATEMENT

Zu‐Cai Xu is an Associate Editor of Ibrain and a coauthor of this article. He was excluded from editorial decision‐making related to the acceptance and publication of this article. Editorial decision‐making was handled independently by the Editor‐in‐Chief to minimize bias.

## ETHICS STATEMENT

Not applicable.

## Data Availability

Not applicable.
